# Downregulation of COP9 signalosome subunits differentially affects the CSN complex and target protein stability

**DOI:** 10.1186/1471-2091-8-27

**Published:** 2007-12-19

**Authors:** Andreas Peth, Christoph Berndt, Wolfgang Henke, Wolfgang Dubiel

**Affiliations:** 1 Department of Surgery, Division of Molecular Biology, Charité – Universitätsmedizin Berlin, Monbijoustrasse 2, D-10117 Berlin, Germany; 2 Department of Otorhinolaryngology, Molecular Biology Research Laboratory, Charité – Universitätsmedizin Berlin, Charitéplatz 1, D-10117 Berlin, Germany

## Abstract

**Background:**

The COP9 signalosome (CSN) is a conserved protein complex in eukaryotic cells consisting of eight subunits (CSN1 to CSN8). Recent data demonstrate that the CSN is a regulator of the ubiquitin (Ub) proteasome system (UPS). It controls substrate ubiquitination by cullin-RING Ub ligases (CRLs), a process that determines substrate specificity of the UPS. The intrinsic deneddylating activity localized to CSN5 as well as the associated kinases and deubiquitinating activity are involved in the regulatory function of CSN. The exact mechanisms are unclear. In this study we knocked down CSN1 (siCSN1), CSN3 (siCSN3) and CSN5 (siCSN5) by specific siRNA oligos permanently expressed in HeLa cells. The analysis and comparison of siRNA cells revealed differential impact of individual subunits on CSN structure and function.

**Results:**

Permanent knockdowns of CSN1 and CSN3 led to a reduction of the subunits to approximately 40%, which is accompanied by a proportional decrease of the CSN holocomplex. In contrast, downregulation of CSN5 in HeLa cells reduced the CSN5 protein below 20% without significant effects on the remaining complex. The CRL component Rbx1 was characterized by accelerated proteolysis in siCSN1 and siCSN3 and also in siCSN5 cells, however, with lesser extent. Immunoprecipitated CSN complex from siCSN5 cells was less effective in phosphorylating c-Jun and p27. Accelerated degradation of c-Jun in siCSN5 cells was rescued by overexpression of CSN5 as well as of the deneddylation mutant CSN5D151N. Overexpression of CSN5 cannot rescue c-Jun destabilization in siCSN1.

**Conclusion:**

There exists a coordinated downregulation of CSN subunits in the CSN1 and CSN3 knockdowns. The underlying regulatory mechanisms are obscure. CSN5 seems to possess a specific status in HeLa cells. Its reduction is not connected with coordinated downregulation of other subunits. CSN knockdowns confirm that the stabilization of the CRL component Rbx1 is a major CSN function. In addition, downregulation of CSN subunits influences the stability of important cellular regulators such as c-Jun and p27.

## Background

The COP9 signalosome (CSN) is a conserved protein complex, which controls eukaryotic protein degradation via the ubiquitin (Ub) proteasome system (UPS) [1,2]. In mammals the core complex consists of 8 subunits (CSN1 to CSN8) [3], the exact function of which is not exactly known. CSN5 exhibits a MPN^+^/JAMM domain [4,5] responsible for metalloprotease activity. As a complex-bound protein CSN5 removes NEDD8, an ubiquitin-like protein, from cullins. This cleavage of an isopeptide bond called deneddylation controls the ubiquitination by cullin-RING Ub ligases (CRLs), a large family of multisubunit E3s [6,7]. Cullin proteins (Cul1 to Cul7) are components of CRLs functioning as scaffolds of the Ub ligase complexes. Cullin neddylation and deneddylation regulates the CRL complex assembly in a substrate dependent manner as it has been shown for Cul1-SCF^Skp2 ^and p27^Kip ^(p27) [8]. In addition, CRLs contain RING-domain proteins, frequently Rbx1 or Rbx2, which are responsible for ubiquitination as well as for neddylation [9]. Substrate specificity of the CRLs is determined by components called substrate receptors including F-box proteins in Cul1-based CRLs and BTB-domain proteins in Cul3-complexes [7].

The CSN is associated with a cysteine protease called Ub specific protease 15 (USP15), which belongs to the family of deubiquitinating enzymes (DUBs) [10,11]. USP15 is able to cleave linear as well as branched Ub chains. The cleavage of lysine 48-linked poly-Ub chains requires a functional Zn finger [10]. The cysteine protease regulates the activity of CRLs by protecting Rbx1 [10] as well as F-box and BTB-domain proteins [11,12] from autoubiquitination and degradation.

In addition, the CSN is associated with kinases such as CK2, PKD [13], Akt [14] and inositol 1,3,4-trisphosphate 5/6 kinase [15]. The kinases modify substrates of the UPS and determine their stability [1]. The CK2 binds to subunits CSN3 and CSN7 whereas the PKD interacts with CSN3 [13]. CSN-associated CK2 phosphorylates the tumor suppressor p53 [16] and the inhibitor of cyclin-dependent kinases p27 [14], which targets the proteins to degradation by the 26S proteasome. In contrast, phosphorylation of c-Jun stabilizes the transcription factor towards the UPS [13,17]. Inhibitors of CSN-associated kinases such as curcumin or emodin elevate the amount of p53, which causes apoptosis in tumor cells [18]. On the other hand, by blocking phosphorylation these compounds target c-Jun to rapid degradation by the UPS [13,18]. Subunit CSN5 directly interacts with c-Jun [19], p27 [20] as well as with p53 [16], which is presumably necessary for CSN-mediated phosphorylation and degradation of the proteins, although the exact interrelations are not yet clear. The impact of permanent downregulation of CSN5 in HeLa cells on the stability of c-Jun and p27 was investigated in the present study.

To analyze functional characteristics of genes in yeast, knockouts have been performed. Unfortunately knockouts of CSN genes in yeast possess only weak or no phenotypes [21]. In contrast, CSN knockouts in *Drosophila *[22], in plants [2,23] and in mammals [24,25] are lethal. Recently functional genomic studies in mammalian cells include specific siRNA oligos that cause degradation of the target gene mRNAs. For example, the conditional knockdown of CSN5 expression in HEK293 cells using a doxycycline-inducible shRNA system led to the degradation of F-box proteins accompanied with reduced CRL activity [26]. It has been shown that downregulation of Jab1/CSN5 in leukemia cell lines prevented p27 degradation [27]. Recent knockdown studies in *Drosophila *revealed a role of the CSN in the cell cycle [28].

By using specific siRNA oligos against CSN1, CSN3 and CSN5 permanently expressed in HeLa cells, we studied the impact of CSN subunit knockdowns on the CSN complex stability and target protein degradation. We demonstrate that knockdowns of CSN1 or CSN3 led to a downregulation of the entire CSN complex, whereas in case of CSN5 downregulation the remaining complex was stable. The decrease of the binding subunit CSN5 in siCSN5 cells as well as diminished CSN complex in siCSN1 and siCSN3 cells led to reduced stability of Rbx1 and c-Jun, whereas p27 was stabilized in siCSN1 cells.

## Results

### The CSN5 knockdown is unique compared to CSN1 or CSN3 knockdowns in HeLa cells

**Figure 1:**
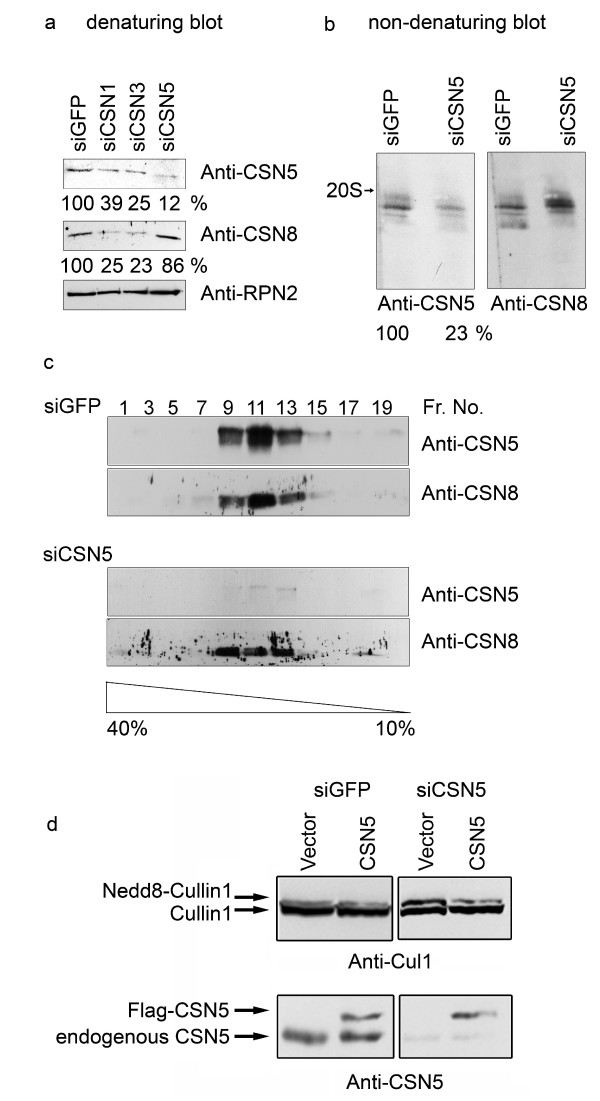
**Comparison of HeLa cells permanently expressing siRNA oligos against CSN1, CSN3 or CSN5**. **(a) **Western blots with lysates from HeLa cells permanently expressing siRNA oligos against GFP (siGFP cells = control cells), CSN1 (siCSN1 cells), CSN3 (siCSN3 cells) and CSN5 (siCSN5 cells) were performed using the anti-CSN5 and the anti-CSN8 antibody. RPN2 was analyzed as a loading control. The signals obtained with the anti-CSN5 and the anti-CSN8 antibodies were evaluated by densitometry. Values obtained in control cells were put to 100%. Similar blots with anti-CSN1 and anti-CSN3 antibodies were published recently [29]. 20S indicates the position of the 20S proteasome in the non-denaturing gels under our conditions. **(b) **Non-denaturing gel electrophoresis and blot with lysates from control cells (siGFP) and siCSN5 cells using the Phast-gel system. The same blot was probed with the anti-CSN5 antibody and the anti-CSN8 antibody. Whereas the anti-CSN5 antibody revealed a reduction to 23% as compared to the control, there was no decrease of the CSN complex observed with the anti-CSN8 antibody. **(c) **Glycerol gradient centrifugation of lysates from siGFP and siCSN5 cells. Aliquots of fractions 1 to 19 were probed by Western blotting using the anti-CSN5 and the anti-CSN8 antibody. Under these conditions the CSN complex sedimented into fractions 9 to 13. Free subunits were not detected. Under these conditions the 20S proteasome sedimented into fraction 5 to 9. **(d) **Control cells (siGFP) and siCSN5 cells were transfected with Flag-CSN5wt. The upper panels demonstrate Western blots with the anti-Cul1 antibody demonstrating two bands, deneddylated and mono-neddylated Cul1. There was significantly more mono-neddylated Cul1 in siCSN5 cells (vector) as compared to siGFP cells. Deneddylation was restored by overexpression of CSN5. The lower panel shows Western blots with the anti-CSN5 antibody visualizing the endogenous and the Flag-tagged exogenous CSN5 protein.

In order to study the influence of individual subunits on CSN composition and functions CSN1, CSN3 as well as CSN5 were downregulated by specific siRNA oligos permanently expressed in HeLa cells. As a control HeLa cells permanently expressing siRNA oligos against GFP (siGFP) were used. Fig. 1a demonstrates the impact of siRNA oligos against CSN1 (siCSN1), CSN3 (siCSN3) and CSN5 (siCSN5) on the protein levels of the corresponding CSN subunits in HeLa cells. The Western blot shows that downregulation of CSN1 and CSN3 is accompanied by a reduction of subunits CSN5 and CSN8. As it has been shown earlier under these conditions CSN1 and CSN3 protein levels were reduced below 40%, which was paralleled by a similar reduction of the holocomplex [29]. In contrast, downregulation of CSN5 below 20% had little effect on the stability of CSN8. Non-denaturing gel electrophoresis and glycerol gradient centrifugation revealed that siCSN5 cells possessed CSN complexes with less or no CSN5 as compared to control cells (Fig. 1b and 1c). In native gels the CSN migrated to the same position as the control holocomplex, however, exhibiting only about 20% of the wild type (wt) CSN5 content (Fig. 1b). In glycerol gradients performed with lysate from siCSN5 cells the CSN5 protein was hardly detectable in all fractions whereas the CSN8 protein was not significantly changed in the common CSN fractions (Fig. 1c). These data indicated that the knockdown of CSN5 had little impact on the total amount and the size of the CSN in HeLa cells. This differs from CSN1 or CSN3 knockdowns where the amount of the CSN holocomplex decreased proportionally to the reduction of the CSN subunits [29]. In siCSN5 cells deneddylation of Cul1 was reduced compared to the control with siGFP cells (see Fig. 1d). There was an increase in mono-neddylated Cul1 as indicated by the Western blot. This effect could be reversed by overexpressing CSN5wt into siCSN5 cells. As shown in Fig. 1d, deneddylation of Cul1 was restored by exogenous CSN5 indicating that the subunit was integrated into the complex.

### CSN knockdowns destabilize the RING-domain protein Rbx1

**Figure 2:**
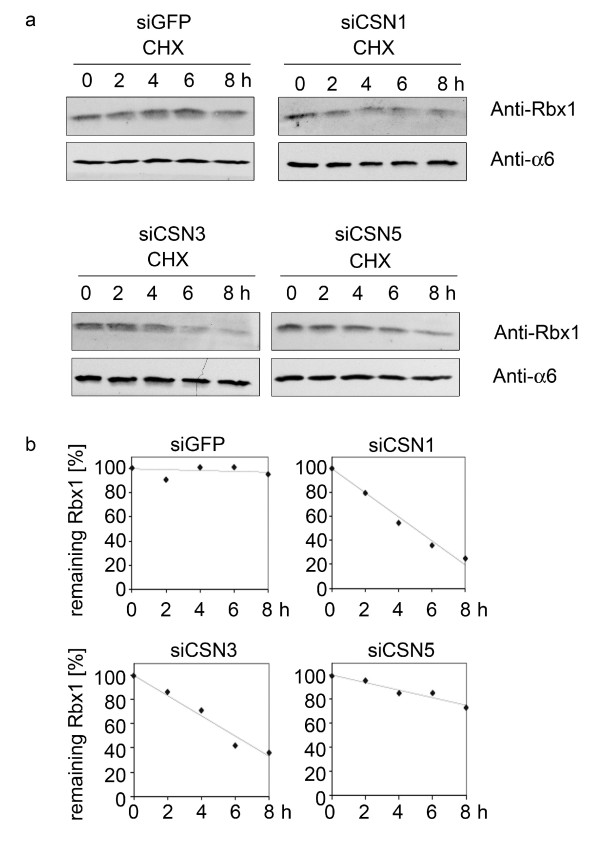
**The CRL component Rbx1 is destabilized in CSN knockdown cells**. **(a) **CHX chase experiments were performed with siGFP, siCSN1, siCSN3 and siCSN5 cells. After the indicated time cell lysates were analyzed by Western blotting using the anti-Rbx1 antibody. The α6 proteasomal subunit was probed as loading control. **(b) **Densitometric analysis of the Rbx1 signals from (a). The densitometric signal at 0 h was put to 100% and the percentage of Rbx1 signals was plotted against the indicated time.

It has been shown before that the CSN, most likely by the CSN-associated deubiquitinating enzyme USP15, protects components of CRLs including the CRL RING-domain component, Rbx1, against autoubiquitination and degradation [10,12]. Therefore, by cycloheximide chase (CHX) experiments we investigated whether downregulation of CSN subunits has any influence on the stability of Rbx1. As shown in Fig. 2a and 2b, in siCSN1, siCSN3 as well as in siCSN5 cells Rbx1 was degraded faster as compared to the control. However, as demonstrated by densitometric analysis the CSN5 knockdown was significantly less effective in stimulating Rbx1 proteolysis as compared to CSN1 and CSN3 knockdowns (Fig. 2b).

### There are differential roles of CSN subunits in affecting target protein stability

The deneddylating MPN^+^-domain protein CSN5 interacts with a large number of ligands. It can be called a substrate receptor of CSN interacting proteins including important regulators such as c-Jun and p27 (for rev. see [1]). In contrast, CSN1 and CSN3 interact with CSN-associated kinases [13,15]. One might postulate that knockdowns of CSN1, CSN3 and CSN5 should cause reduced CSN-mediated phosphorylation of c-Jun and of p27, however, due to different reasons.

**Figure 3:**
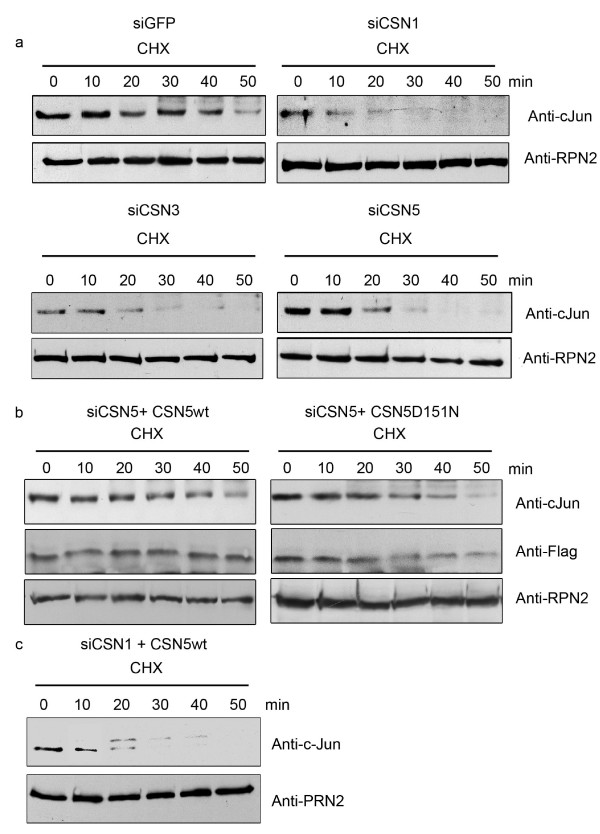
**The degradation of c-Jun is accelerated in CSN knockdown cells**. **(a) **CHX chase experiments were performed with siGFP, siCSN1, siCSN3 and siCSN5 cells. After indicated time aliquots of cell lysates were analyzed by Western blotting using the anti-c-Jun antibody. The same samples were probed with the antibody against the 26S proteasome base subunit RPN2/S1 as a loading control. **(b) **CSN5 knockdown cells were transfected with CSN5wt as in Fig. 1d or with CSN5D151N and 24 h after transfection CHX chase experiments were carried out as in (a). The middle panel shows Western blots with the anti-Flag antibody visualizing the expressed CSN5wt or CSN5D151N proteins **(c) **CSN5wt was overexpressed in siCSN1 cells and after 24 h CHX experiments were performed as in (a). In all CHX experiments a band just above c-Jun appeared after 20 – 30 min, which cross-reacted with the anti-c-Jun antibody (in some blots it was cut off). This protein might be a modified c-Jun. The nature of this modification is currently unknown. Both the putative modified c-Jun as well as unmodified c-Jun disappeared during the experiment.

C-Jun is stabilized by CSN-mediated phosphorylation towards the UPS [13]. To see whether c-Jun stability is changed in siCSN1, siCSN3 and in siCSN5 cells, we measured the proteolysis of the transcription factor in CHX experiments. We expected an accelerated degradation in HeLa cells with downregulated CSN subunits. As shown in Fig. 3a, c-Jun degradation is significantly faster in siCSN1, siCSN3 as well as in siCSN5 cells as compared to the control. The half-life of c-Jun declined from approximately 40 min in the control down to 20 min in CSN1 knockdown cells. The changes in siCSN5 cells might be the result of the reduced CSN5 substrate receptor, which is necessary for c-Jun binding and subsequent phosphorylation. To prove this hypothesis siCSN5 cells were transfected with CSN5wt as in Fig. 1d. As shown in Fig. 3b, left panel, overexpression of CSN5wt increases c-Jun half-life to a level, which is similar to that in siGFP cells (Fig. 3a, upper left panel). Then we asked whether the deneddylating activity of CSN5 is necessary for c-Jun stabilization. For this purpose the CSN5D151N mutant, which lost its metalloprotease activity, was transfected into siCSN5 cells. As seen in Fig. 3b, right panel, overexpression of the CSN5D151N mutant rescued c-Jun just like CSN5wt, indicating that the mutant supports binding of c-Jun to the CSN and perhaps subsequent phosphorylation. In siCSN1 and in siCSN3 cells overexpression of CSN5wt should not block accelerated degradation of c-Jun. This was tested with siCSN1 cells and is shown in Fig. 3c.

**Figure 4:**
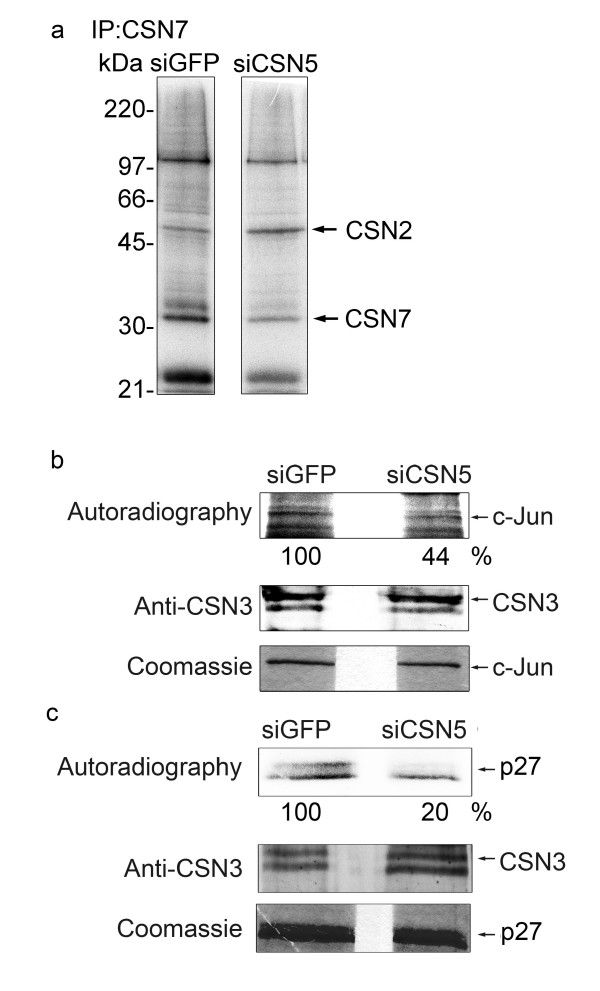
**Phosphorylation of c-Jun and p27 by the CSN immunoprecipitated from siCSN5 cells**. **(a) **Autophosphorylation of CSN subunits CSN2 and CSN7 with CSN immunoprecipitates from control cells (siGFP) and from siCSN5 cells. The CSN was precipitated with the anti-CSN7 antibody. The immunoprecipitate was incubated with ^32^P-γ-ATP, separated by SDS-PAGE and the dried gel was exposed to X-ray films. The autoradiography shows phosphorylation of CSN2 and CSN7 in the precipitate from siGFP as well as siCSN5 cells. Although same numbers of cells were used and the amount of the CSN complex is almost identical in control and siCSN cells, there was a slight increase of CSN2 and a decrease of CSN7 and overall phosphorylation with the CSN from siCSN5 cells. The exact reasons for these differences are not known at the moment. **(b) **Immunoprecipitates from siGFP or siCSN5 cells were used to phosphorylate c-Jun. The upper panel shows the phosphorylation of c-Jun by autoradiography demonstrating a decrease of c-Jun phosphorylation by more than 50% as estimated by densitometry. In the middle panel aliquots were analyzed by Western blotting using the anti-CSN3 antibody to control that equal amounts of the CSN were immunoprecipitated. The lower panel shows the Coomassie stain of c-Jun indicating that the same amounts of the protein were used for kinase assays. **(c) **The same experiments as performed with c-Jun (see b) were carried out with p27. As seen in the autoradiography the phosphorylation of p27 is significantly reduced with the CSN from siCSN5 cells as compared to the control.

To analyze whether cellular CSN lost its ability to phosphorylate c-Jun or p27 in siCSN5 cells, the CSN was immunoprecipitated from siGFP and siCSN5 cells with the anti-CSN7 antibody and kinase assays were performed with the precipitates. As shown in Fig. 4a, autophosphorylation of CSN2 and of CSN7 was detected indicating that the CSN-associated kinases precipitated from siCSN5 cells were active. It seemed that overall phosphorylation was slightly reduced, although autophosphorylation of CSN2 increased. The phosphorylation of recombinant c-Jun was diminished by more than 50% with the precipitate from siCSN5 cells as compared with the control (Fig. 4b). The effect is even more obvious with recombinant p27. Almost no phosphorylation was observed with the immunoprecipitate from siCSN5 cells (Fig. 4c).

**Figure 5:**
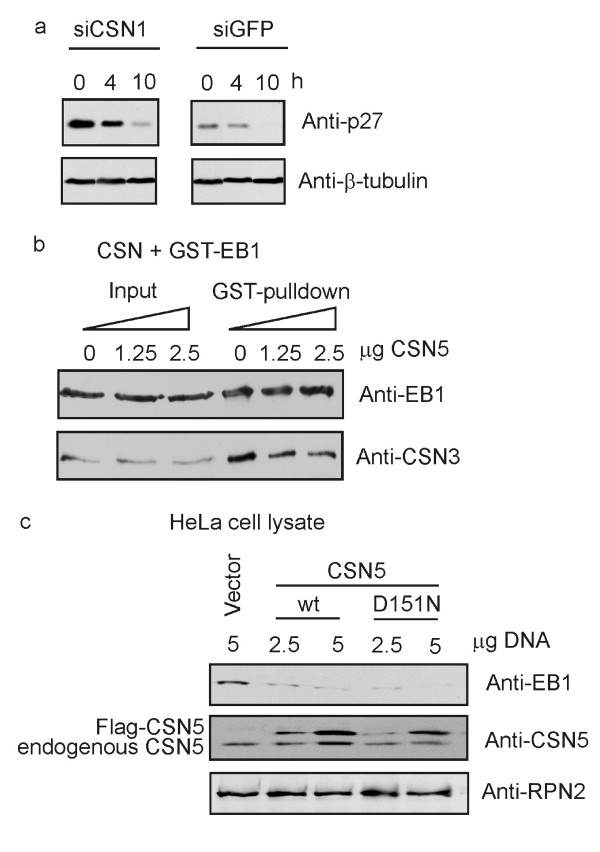
**Binding of p27 and of EB1 to the CSN determines the stability of the two cellular regulators**. **(a) **Steady state levels of p27 were determined by Western blotting in siCSN1 and siGFP HeLa cells, which were synchronized as described before [14]. After reentry into G1 phase Western blots were performed with aliquots from the cytoplasm obtained after 0, 4 and 10 h. The cytoplasmic fraction was prepared as described using SOS protein as a marker for the cytoplasm [14]. As a loading control β-tubulin was analyzed. At the beginning of G1 phase the cell cycle inhibitor p27 was stabilized in the cytoplasm of CSN1 knockdown cells as compared to control cells (siGFP). **(b) **Purified CSN, recombinant GST-EB1 and increasing amounts of recombinant His-CSN5 were incubated for 30 min at 37°C. After incubation the mixture was directly analyzed by Western blotting (Input) or GST-pulldowns were performed and the precipitates were probed by anti-EB1 and anti-CSN3 antibodies. With increasing amounts of His-CSN5 less CSN complex was pulled down with GST-EB1 indicating that the binding between EB1 and the CSN was disturbed by CSN5. **(c) **The impact of the overexpression of CSN5wt or CSN5D151N mutant on EB1 steady state levels in HeLa cells. Transfection with the empty vector was used as control. Cells were lyzed 24 h after transfection and aliquots were analyzed by Western blotting using the anti-EB1 antibody (upper panel). The same lysates were tested with the anti-CSN5 antibody showing the endogenous and the Flag-tagged exogenous CSN5 protein (middle panel). The proteasome subunit RPN2/S1 was probed as a loading control (lower panel).

The above data indicate that downregulation of CSN5 led to a reduced phosphorylation of c-Jun by the immunoprecipitated CSN (Fig. 4b), which correlated with an accelerated degradation of the transcription factor (Fig. 3a). Since CSN-mediated phosphorylation of p27 accelerates its degradation by the UPS [14], we expected a stabilization of p27 in cells with CSN subunit knockdowns. Therefore, siCSN1 cells were synchronized by serum-deprivation for 24 h and then driven into G1 phase of cell cycle by serum complementation as performed before [14]. As seen in Fig. 5a, there is a significant stabilization of p27 after serum starvation (0 h) in the cytoplasm as compared to the control, which is most likely due to a reduced degradation in siCSN1 cells during starvation. Ten hours after reentry into the G1 phase the cell cycle inhibitor was still detectable in siCSN1 cells, whereas it disappeared in siGFP cells.

A decreased phosphorylation in siCSN1 cells has been shown for the microtubule-end-binding protein 1 (EB1) [29]. Just like c-Jun the protein binds to CSN5 and is stabilized by CSN-mediated phosphorylation. By *in vitro *pulldowns with purified CSN and recombinant GST-EB1 and by adding increasing amounts of recombinant CSN5 it was demonstrated that EB1 can be competed away from its CSN5 binding site (Fig. 5b). A similar experiment was performed in cells. CSN5wt or CSN5D151N mutant was overexpressed in HeLa cells and the binding and phosphorylation of endogenous EB1 to the CSN was indirectly estimated by EB1 stability. As seen in Fig. 5c, overexpression of CSN5wt led to an accelerated degradation of EB1, most likely because it was competed away from the CSN and was less phosphorylated. The same effect was obtained by overexpressing CSN5D151N indicating that the metalloprotease activity of CSN5 is not necessary for this competition.

## Discussion

In the present study we compared the effects of CSN subunit downregulation on the structure and functions of the holocomplex. The data presented here as well as in our recent work on EB1 [29] demonstrate that downregulation of CSN1 and CSN3 by specific siRNA oligos permanently expressed in HeLa cells led to a proportional downregulation of the CSN holocomplex. In other words, knockdowns of CSN1 and CSN3 cause a proportional reduction of all determined CSN subunits paralleled by a decrease of the holocomplex. None of the CSN subunits were detected as free proteins under these conditions. Similar data were obtained by Kato and co-workers in murine embryonic fibroblasts were a reduction in the level of CSN3 protein with siRNA decreased the total amount of the holocomplex [30]. The complete deletion of the CSN3 subunit in mice and also in other higher eukaryotes is lethal, which presumably can be due to the complete disappearance of the CSN complex [24]. These data indicate that in mammalian cells exists a coordinated up and down regulation of CSN subunits accompanied by an assembly or disassembly process of the holocomplex.

On the contrary, downregulation of subunit CSN5 had little or no effect on other CSN subunits and did not lead to the proportional disappearance of the residual complex. Similar effects were observed by downregulation of CSN5 in *Drosophila *as well as in HeLa cells [31,32]. In other words, in siCSN5 cells CSN complexes possess less or no CSN5. This might indicate that CSN5 has a different impact on the coordinated assembly of the CSN as compared to CSN1 and CSN3. On the other hand, a complete depletion of CSN5 in *Arabidopsis *resulted in CSN instability and the decay of various CSN components [33]. Interestingly, overexpressed CSN5wt is most likely integrated into the CSN complex in siCSN5 cells, because it is able to restore deneddylation activity of the particle (see Fig. 1d).

Although CSN1, CSN3 as well as CSN5 knockdowns led to destabilization of Rbx1, the effects are different. Whereas in siCSN1 and siCSN3 cells Rbx1 degradation is significantly accelerated, in siCSN5 cells the RING-domain protein is less destabilized (see Fig. 2). The protection of Rbx1 from autoubiquitination and degradation is mostly due to the associated deubiquitinating enzyme USP15, which interacts predominantly via CSN7 with the CSN [10]. This might explain why the stability of Rbx1 in siCSN5 cells is less affected as compared to siCSN1 or siCSN3 cells. Our siRNA studies confirm the hypothesis that the CSN stabilizes CRL components by protecting them against autoubiquitination [10,12]. With less CSN and its associated USP15 in cells there is less protection of the CRLs.

We speculate that changed stabilities of c-Jun, p27 and EB1 in siCSN1, siCSN3 as well as siCSN5 cells can be due to reduced CSN-mediated phosphorylation of these proteins. In siCSN1 and siCSN3 cells the amount of the CSN platform itself together with its associated kinases was decreased. In CSN5 knockdown cells the reduction of the substrate receptor CSN5 might be sufficient to cause a diminished phosphorylation of the tested substrates (see Fig. 4). Immunoprecipitated CSN from siCSN5 cells was less efficient in phosphorylating c-Jun as well as p27 as compared to the control. This effect can be explained by the reduction of CSN5 and, at least in part, by reduced overall kinase activity (see Fig. 4a) perhaps caused by a changed CSN structure. In contrast to siCSN1 cells the accelerated degradation of c-Jun in siCSN5 cells was blocked by overexpression of CSN5wt as well as of CSN5D151N mutant (see Fig. 3). Based on these data we came to two conclusions. (i) The overexpressed CSN5wt was integrated into CSN5-deficient CSN complexes and acts as substrate receptor allowing normal substrate phosphorylation. (ii) The rescue of c-Jun stability by overexpressing CSN5D151N demonstrates that the mutant is as potent as the CSN5wt indicating that the metalloprotease activity of CSN5 was not responsible for the effect.

Presumably reduced phosphorylation of p27 in CSN knockdown cells resulted in a stabilization of the cell cycle inhibitor during serum starvation (see Fig. 5a). Thus, the CSN might control the ubiquitination of p27 by two different activities. First, it regulates the assembly of the appropriate Cul1-CRL complex for p27 ubiquitination by deneddylation [8]. Second, it seems to be a platform for p27 phosphorylation that most likely accelerates p27 ubiquitination.

Our data also show that one should be cautioned with the overexpression of CSN5. In HeLa cells free CSN5 is undetectable under our conditions. Overexpression of CSN5 produces a free CSN5 protein pool, which stimulates the degradation of EB1. As shown by competition experiments in Fig. 5b and 5c, this is most likely due to the trapping of EB1 by free CSN5 and, as a result, to reduced CSN-mediated phosphorylation and destabilization of EB1. We speculate that overexpression of CSN5 might exert artificial effects on other substrates that interact with CSN5 as well.

Future knockdown experiments with other CSN subunits are necessary to fully understand the relationship between structure and function of the CSN complex. In addition, the integration of diverse mutated subunits into the CSN complex where possible will elucidate specific functions of subunit domains.

## Conclusion

We have shown that downregulation of CSN1 and CSN3 reduced the CSN holocomplex. In contrast, downregulation of CSN5 did not change the remaining CSN complex, although CSN5 protein was reduced. All studied knockdown cells were characterized by a destabilization of the CRL component Rbx1 and of the transcription factor c-Jun. In CSN5 knockdowns c-Jun destabilization was rescued by CSN5 overexpression, demonstrating the substrate receptor role of CSN5.

## Methods

### Preparation of the CSN, cell culture and siRNA knockdown of CSN subunits

The human CSN complex was purified from red blood cells as outlined in detail [34]. HeLa cells were cultured as described previously [16]. siRNA-mediated knockdown of CSN1 and CSN3 was performed as outlined recently [29] using the pSUPER system. The knockdown of CSN5 was performed with the target sequences published by Groisman and co-workers [32]. Synchronization of HeLa cells was carried out as described previously [14].

### Transfection of HeLa cells, site directed mutagenesis and CHX chase experiments

HeLa cells were transfected with Lipofectamine2000 (Invitrogen) according to the manufacturers recommendations. The metalloprotease mutant CSN5D151N was created by site directed mutagenesis as outlined before [10]. Expression of Flag-CSN5 and of Flag-CSN5D151N in HeLa cells was performed as described before [17]. Rescue experiments of c-Jun degradation were performed after transfection with 2.5 μg of CSN5wt DNA or 5 μg of CSN5D151N DNA. CHX chases were carried out at a final concentration of 20 μg/ml CHX as in [29].

### Immunoprecipitation, kinase assays, Western blotting, competition experiments and glycerol gradients

Immunprecipitations and glycerol gradient centrifugations were performed as described [29]. The *in vitro *kinase assays were carried out as outlined previously [13]. SDS-PAGE, Western blotting and ECL detection (GE Healthcare) were performed by standard procedures. Non-denaturing electrophoresis was carried out using the Phast-System (GE Healthcare). Competition experiments with EB1, CSN5 and the CSN complex were performed as described [29] using recombinant GST-EB1, recombinant His-CSN5 and purified CSN. In this study the following antibodies were used: Anti-CSN5 (provided by B. Christy), anti-Rbx1 (Zymed), anti-CSN8 (Biomol), anti-α 6 and anti-RPN2 (provided by C. Hendil), anti-c-Jun (Upstate), anti-p27 and anti-EB1 (Santa-Cruz), anti-β-tubulin (Covance), anti-CSN3 antibodies [35] and anti-Flag (Sigma).

## List of abbreviations

CSN: COP9 signalosome

Ub: ubiquitin

UPS: ubiquitin proteasome system

CRL: cullin-RING ubiquitin ligases

p27^Kip^: p27

CHX: cycloheximide

## Authors' contributions

Most of the experiments were performed by AP. The synchronization of HeLa cells and the estimation of p27 in siCSN1 cells were carried out by CB. The CSN5 knockdown cells were established by WH. AP and WD conceived and designed the experiments and wrote the manuscript. All authors read and approved the final manuscript.
